# Supplementary material for: Bacteriophages Roam the Wheat Phyllosphere
Source: Viruses. 2022 Jan 26;14(2):244. doi: 10.3390/v14020244 (PMC8876510; doi:10.3390/v14020244)
Supplement: Supplementary file 1 [file viruses-14-00244-s001.zip › Supplementary_Bacteriophagesroam_phyllosphere 2.pdf]

Bacteriophages roam the wheat phyllosphere

Laura Milena Forero-Junco[1,*], Katrine Wacenius Skov Alanin[1,2], Amaru Miranda Djurhuus[1], Witold Kot[1*], Alex Gobbi[1], & Lars Hestbjerg Hansen[1*]

Supplementary figures and tables

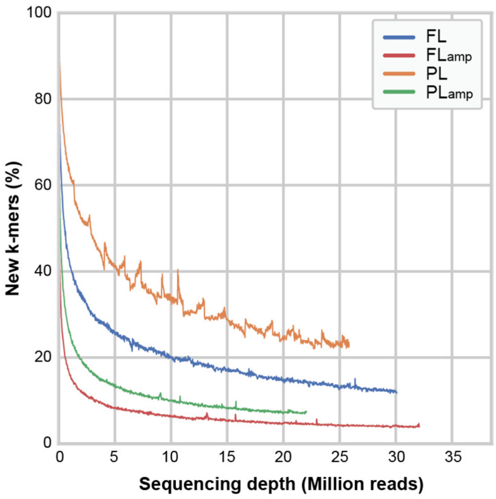

Figure S1. K-mer rarefaction curve. The fraction of new k-mers, equivalent to unique reads, is plotted as a function of the number of reads per sample.

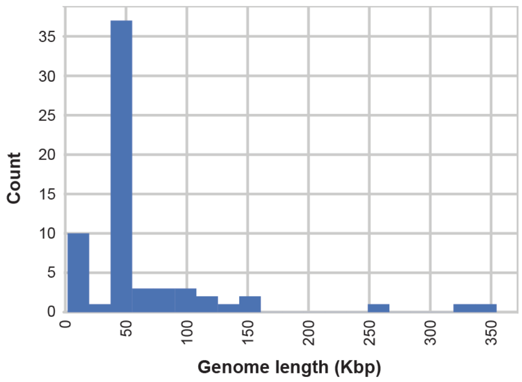

Figure S2. Length distribution of the predicted complete genomes.

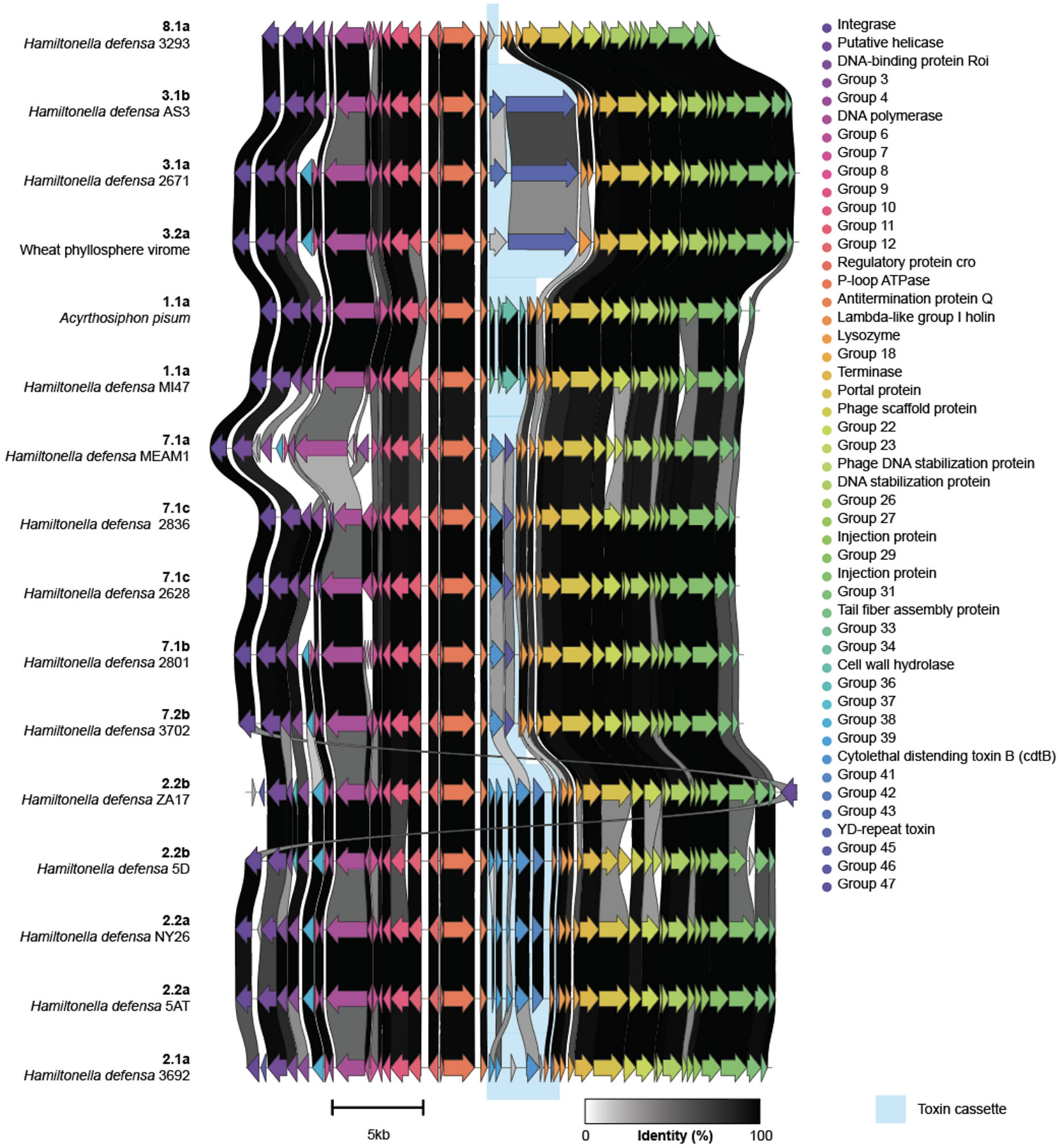

Figure S3. **New APSE-phage variant discovered in the wheat phyllosphere.** Genomic comparisons of representative variants in the APSE group at the amino acid level (clinker). The main variation between these members is observed in the region encoding a toxin cassette, carrying either Shiga-like toxins, cytolethal distending toxins (CdtB), or YD-repeat toxins, which characterizes APSE variants. The wheat APSE vOTU resembles members of variant 3, encoding a YD-repeat toxin. However, with only 55% amino acid identity.

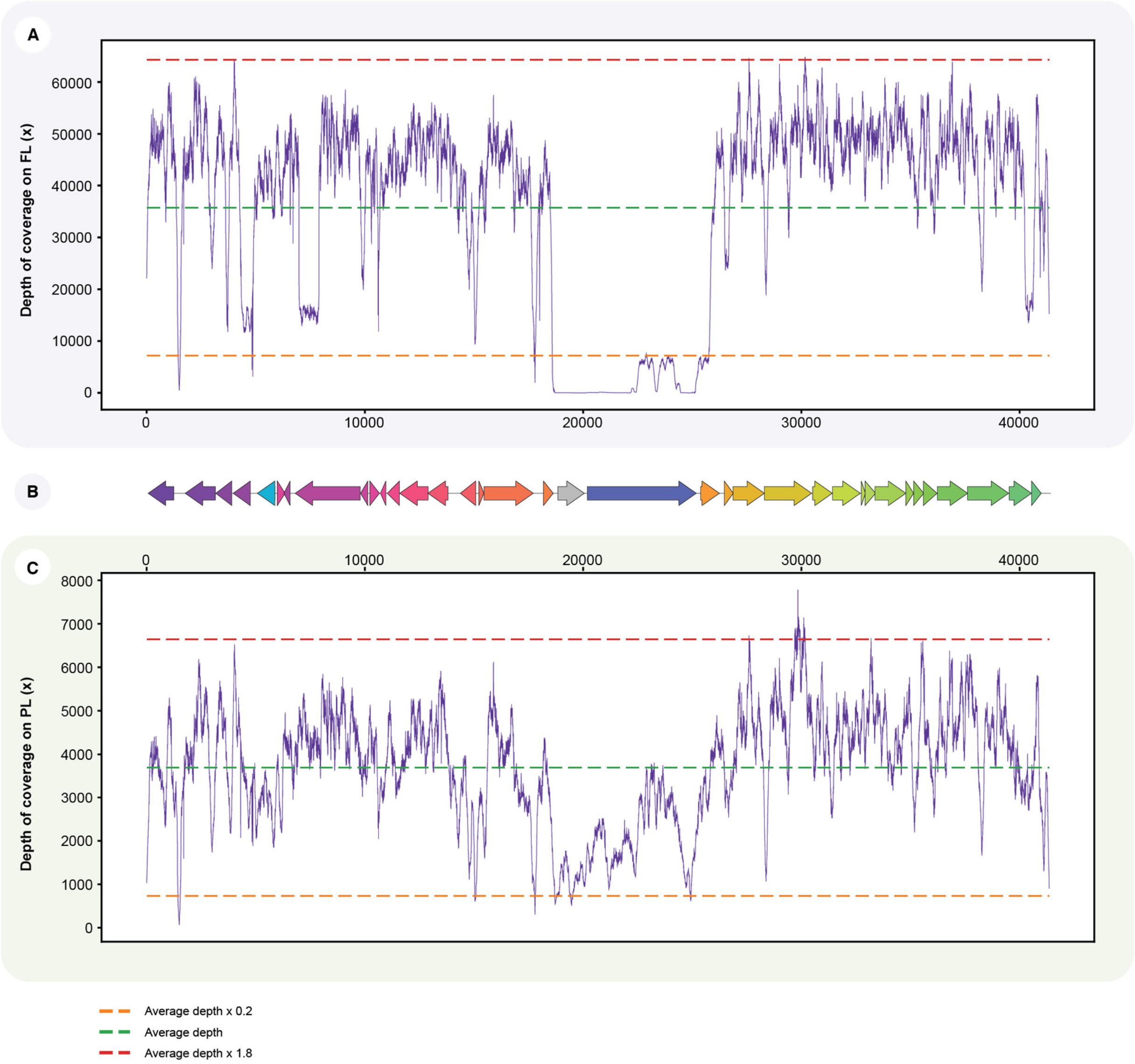

Figure S4. The wheat-phyllosphere APSE population is differently represented in the two wheat viromes. The read coverage of the viromes extracted from the penultimate leaf (**A**) and flag leaf (**C**) varies greatly in the region of the APSE phage genome (**B**) encoding the putative toxin cassette. The variant represented by the wheat vOTU, encoding a YD-repeat toxin (typical of the APSE-3 variants) only has coverage across the putative toxin cassette region in the flag leaf virome, revealing a great deal of variation in the toxin-encoded region across both the penultimate and flag leaf viromes.

Table S1: **The DNA concentration of six different sample types.** Overview of DNA concentration from the microbial and virus fraction of both leaf types, as well as the DNA concentration of the amplified virus fraction.

Sample	DNA conc. (ng/µl)	Sample size (µl)	Total amount of DNA	Raw sequencing reads (paired)
FL_{mf}	100	100	10 µg	20,347,654
PL_{mf}	8	100	800 ng	20,392,188
FL virome	4.58	20	91.6 ng	71,400,921
PL virome	1.86	20	37.2 ng	72,460,435
FL_{mda} virome	5.28	10	52.8 ng	35,481,660
PL_{mda} virome	1.22	10	12.2 ng	24,508,019

Table S2: **The relative abundance of the bacterial community and the number of linked vOTUs.** Relative abundance of the microbial fraction at family level. Reads were classified with Kraken at family level for the FL, PL, and combined reads. The last column corresponds to the number of vOTUs that were linked to bacterial families by matching viral sequences with bacterial CRISPR spacers.

Bacterial taxonomy (family)	FL relative abundance	PL relative abundance	Combined relative abundance	vOTUs matched to bacterial host
Xanthomonadaceae	17.59%	46.31%	33.21%	21
Pseudomonadaceae	32.20%	19.92%	25.12%	78
Sphingobacteriaceae	9.00%	3.91%	6.19%	3
Erwiniaceae	4.60%	6.51%	5.64%	40
Oxalobacteraceae	7.16%	4.03%	5.43%	2
Sphingomonadaceae	2.99%	3.16%	3.07%	23
Flavobacteriaceae	1.89%	3.77%	2.90%	3
Yersiniaceae	3.10%	1.10%	1.98%	16
Microbacteriaceae	2.53%	0.86%	1.61%	2
Enterobacteriaceae	1.44%	0.46%	0.93%	153
Comamonadaceae	0.54%	1.00%	0.79%	9
Hymenobacteraceae	0.37%	1.07%	0.74%	14
Rhizobiaceae	0.51%	0.79%	0.66%	1
Burkholderiaceae	0.47%	0.42%	0.44%	5
Alcaligenaceae	0.36%	0.33%	0.34%	7
Paenibacillaceae	0.51%	0.18%	0.33%	10
Hafniaceae	0.06%	0.45%	0.27%	4
Moraxellaceae	0.57%	0.00%	0.27%	15
Streptomycetaceae	0.15%	0.17%	0.16%	20
Bradyrhizobiaceae	0.09%	0.10%	0.09%	3
Rhodobacteraceae	0.08%	0.11%	0.09%	12

Chitinophagaceae	0.09%	0.08%	0.09%	2
Cytophagaceae	0.07%	0.09%	0.08%	4
Bacillaceae	0.10%	0.04%	0.07%	7
Pectobacteriaceae	0.08%	0.04%	0.06%	13
Mycobacteriaceae	0.05%	0.07%	0.06%	1
Carnobacteriaceae	0.11%	0.00%	0.06%	1
Methylobacteriaceae	0.05%	0.06%	0.06%	2
Rhodospirillaceae	0.04%	0.05%	0.05%	4
Micromonosporaceae	0.00%	0.04%	0.04%	13
Pseudonocardiaceae	0.00%	0.04%	0.04%	3
Rhodanobacteraceae	0.00%	0.03%	0.03%	3
Acetobacteraceae	0.00%	0.00%	0.03%	6
Halomonadaceae	0.00%	0.00%	0.03%	13
Vibrionaceae	0.00%	0.00%	0.03%	5
Chromobacteriaceae	0.00%	0.00%	0.03%	5
Micrococcaceae	0.00%	0.00%	0.03%	2
Clostridiaceae	0.00%	0.00%	0.02%	3
Hyphomicrobiaceae	0.00%	0.00%	0.02%	1
Campylobacteraceae	0.00%	0.00%	0.02%	2
Cyclobacteriaceae	0.00%	0.00%	0.02%	1
Zoogloeaceae	0.00%	0.00%	0.02%	1
Morganellaceae	0.00%	0.00%	0.02%	5
Aeromonadaceae	0.00%	0.00%	0.02%	2
Alteromonadaceae	0.00%	0.00%	0.02%	2
